# The small GTPase RhoG mediates glioblastoma cell invasion

**DOI:** 10.1186/1476-4598-11-65

**Published:** 2012-09-11

**Authors:** Aneta Kwiatkowska, Sebastien Didier, Shannon Fortin, Yayu Chuang, Timothy White, Michael E Berens, Elisabeth Rushing, Jennifer Eschbacher, Nhan L Tran, Amanda Chan, Marc Symons

**Affiliations:** 1 Center for Oncology and Cell Biology, The Feinstein Institute for Medical Research at North Shore-LIJ, Manhasset, NY, USA; 2 Departments of Molecular Medicine and Neurosurgery, Hofstra-North Shore LIJ School of Medicine, Hempstead, NY, USA; 3 Cancer and Cell Biology Division, The Translational Genomics Research Institute, Phoenix, AZ, USA; 4 Cancer Biology Graduate Interdisciplinary Program, University of Arizona, Tucson, AZ, USA; 5 UniversitätsSpital Zürich, Zürich, Switzerland; 6 Barrow Neurological Institute, Phoenix, AZ, USA; 7 The Feinstein Institute for Medical Research 350 Community Dr, Manhasset, NY, 11030, USA

**Keywords:** RhoG, Rac1, cMet, EGFR, Glioblastoma, Invasion

## Abstract

**Background:**

The invasion of glioblastoma cells into regions of the normal brain is a critical factor that limits current therapies for malignant astrocytomas. Previous work has identified roles for the Rho family guanine nucleotide exchange factors Trio and Vav3 in glioblastoma invasion. Both Trio and Vav3 act on the small GTPase RhoG. We therefore examined the role of RhoG in the invasive behavior of glioblastoma cells.

**Results:**

We found that siRNA-mediated depletion of RhoG strongly inhibits invasion of glioblastoma cells through brain slices *ex vivo*. In addition, depletion of RhoG has a marginal effect on glioblastoma cell proliferation, but significantly inhibits glioblastoma cell survival in colony formation assays. We also observed that RhoG is activated by both HGF and EGF, two factors that are thought to be clinically relevant drivers of glioblastoma invasive behavior, and that RhoG is overexpressed in human glioblastoma tumors versus non-neoplastic brain. In search of a mechanism for the contribution of RhoG to the malignant behavior of glioblastoma cells, we found that depletion of RhoG strongly inhibits activation of the Rac1 GTPase by both HGF and EGF. In line with this observation, we also show that RhoG contributes to the formation of lamellipodia and invadopodia, two functions that have been shown to be Rac1-dependent.

**Conclusions:**

Our functional analysis of RhoG in the context of glioblastoma revealed a critical role for RhoG in tumor cell invasion and survival. These results suggest that targeting RhoG-mediated signaling presents a novel avenue for glioblastoma therapy.

## Background

Rho family small GTPases are key signaling elements that control the malignant behavior of cancer cells [1]. In particular, a number of these GTPases have been shown to regulate cell proliferation, survival and invasion [2,3]. Most Rho GTPases act like molecular switches that are active when bound to GTP and inactive when bound to GDP [4]. Their activation is catalyzed by guanine nucleotide exchange factors (GEFs) that induce nucleotide release, allowing for the more abundant cytoplasmic GTP to be loaded onto the GTPase. GTPases are inactivated by GTPase activating proteins (GAPs), which stimulate intrinsic GTP hydrolysis.

Glioblastoma multiforme (grade IV astrocytoma) is one of the most aggressive of all human cancers, with a median survival time of little more than one year [5]. Despite aggressive treatment and recent clinical and molecular advances, the overall survival of glioblastoma patients has not significantly improved over the past two decades. Malignant gliomas, in addition to a core mass, display extensive infiltration of individual cells into the normal brain parenchyma [6]. These invading cells are highly resistant to radiation and chemotherapy, thereby leading to recurrence [7,8]. Furthermore, although anti-angiogenic therapy for recurrent glioblastoma using bevacizumab increases patient survival, it also increases tumor invasiveness [9]. Thus, tumor dispersal is a critical problem in the treatment of these brain tumors and currently, there are no anti-invasive therapies available.

Thus far, the only Rho family GTPases that have been functionally characterized in glioblastoma cells are Rac1, and to a lesser extent, Rac3 [10-12]. Thus, we have shown that Rac1 regulates glioblastoma cell invasion and proliferation [10,11]. In addition, plasma membrane localization of Rac1 in glioblastoma cells *in situ* indicates that Rac1 is constitutively active in a subset of these tumors [12]. We also have identified roles for the Rho family GEFs Trio and Vav3 in the invasive behavior of glioblastoma cells [12]. Both Trio and Vav3 act on the small GTPase RhoG [13,14]. RhoG remains one of the less characterized Rho family members and its role in tumor cells is largely unexplored. RhoG was identified as a growth factor response gene [15] and has also been shown to regulate cell migration, neurite outgrowth and cell survival [16-18]. The signaling elements, including receptors and GEFs, that mediate RhoG activation largely remain to be identified [19].

In this paper, we focus on the role of RhoG in the invasive behavior of glioblastoma cells. We show that RhoG is critical for glioblastoma cell invasion, both *in vitro* and in *ex vivo* brain slices. We also demonstrate that RhoG mediates signaling that is stimulated by cMet and EGFR, two receptors that are deregulated in glioblastoma tumors and that RhoG is overexpressed in human glioblastoma tumor tissue versus non-neoplastic brain.

## Results

### RhoG mediates glioblastoma cell invasion

**Figure 1:**
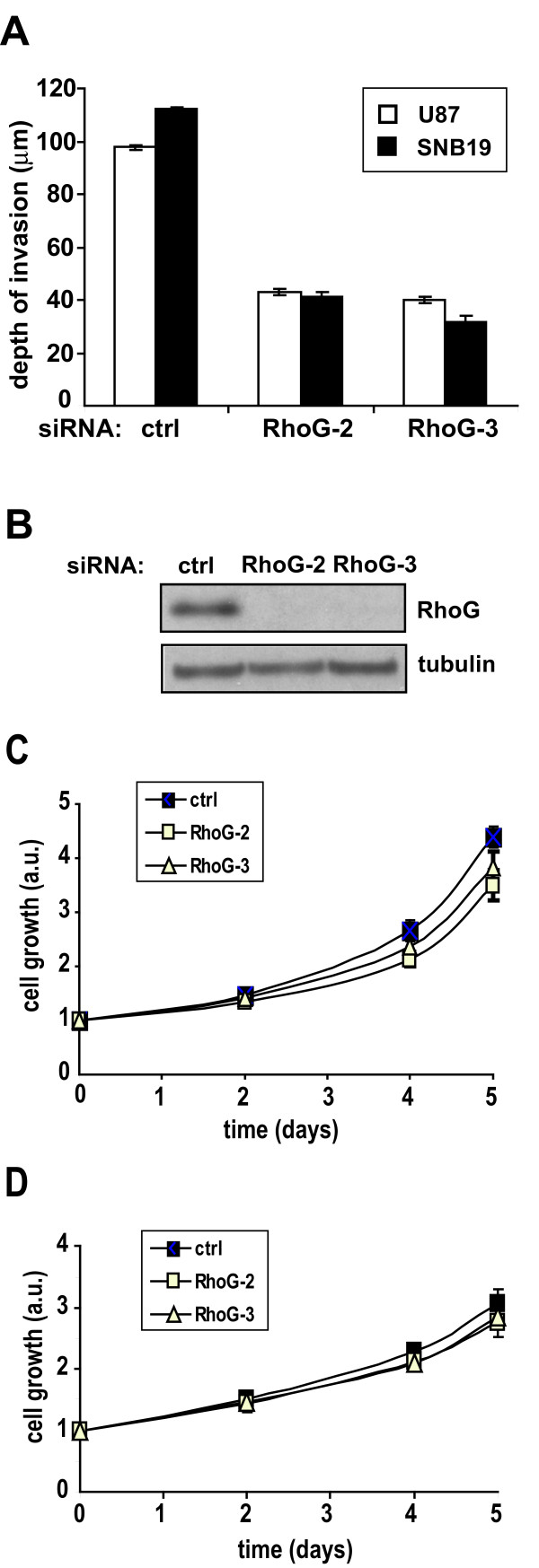
** RhoG is necessary for glioblastoma cell invasion. A**) Brain slice invasion assay. GFP-expressing SNB19 and U87 glioblastoma cells were transfected with siRNAs (10 nM) directed against luciferase (control) or RhoG (two different oligos). The brain slice invasion assay was performed as described in Materials and Methods. Shown are the mean (+/−SEM) of three independent experiments, each performed in triplicate. Differences between RhoG knockdown and control are significant (p < 0.001, two-tailed *t* test). **B**) Western blot demonstrating depletion of RhoG in SNB19 cells. **C** and **D**) Depletion of RhoG has only a marginal inhibitory effect on cell proliferation. SNB19 cells were transfected with siRNAs (10 nM) directed against luciferase (control, solid squares) or RhoG (RhoG-2, empty squares; RhoG-3, empty triangles). Cell proliferation was quantified using the Sulphorhodamine B assay as described in Materials and Methods, either in the presence of 10% FBS (**C**) or in the absence of serum (**D**). Results shown represent the mean +/− SEM for respectively 6 independent experiments (**C**) and 4 independent experiments (**D**).

To examine the role of RhoG in glioblastoma invasion we first determined the effect of depleting RhoG on the invasion of glioblastoma cells into rodent brain slices, a well-established organotypic model [20]. We found that siRNA-mediated depletion of RhoG, using two independent oligos to minimize the risk of RNA off-target effects, strongly impairs invasion of two glioblastoma cell lines, SNB19 and U87, into brain tissue (Figure 1A). Both siRNAs strongly inhibit RhoG expression (Figure 1B).

As RhoG has been shown to promote the proliferation of neural progenitor cells in the mouse cerebral cortex [21], we also examined whether RhoG contributes to the proliferative behavior of glioblastoma cells, as this could be a possible confounding factor in the evaluation of glioblastoma cell invasiveness. We observed that depletion of RhoG has a small inhibitory effect on the proliferation of glioblastoma cells over a 5 day period in the presence of serum, but does not affect cell proliferation over the time period in which the brain slice invasion experiments are carried out (2 days) (Figure 1C). In addition, depletion of RhoG has no significant effect on cell proliferation in the absence of serum (Figure 1D).

### RhoG regulates glioblastoma cell invasion using both Rac1-dependent and Rac1-independent mechanisms

**Figure 2:**
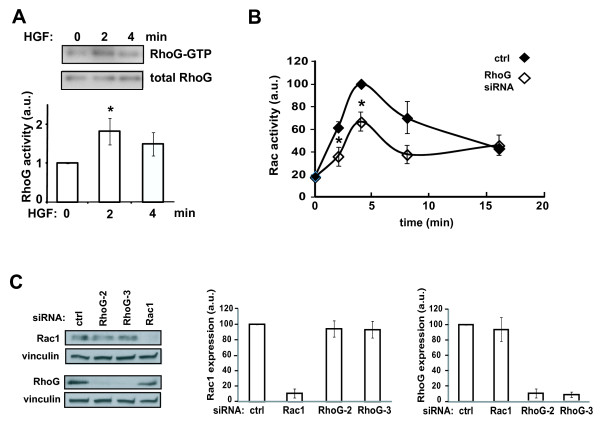
** RhoG mediates HGF-induced Rac1 activation. A**) Time-dependence of RhoG activation by HGF. SNB19 cells were serum starved for 24 hours, and incubated further with or without HGF for the indicated time. Subsequently, cells were lysed and RhoG activity was determined using an active RhoG pull down assay as detailed in Materials and Methods. Western blot shows activated RhoG and total RhoG for HGF (25 ng/ml)-stimulated and control cells. Histogram shows quantification (+/− SEM) of at least 5 independent experiments. **B**) RhoG mediates HGF-induced Rac activation. Cells were transfected with siRNAs (10 nM) directed against luciferase (control) or RhoG-2. Forty eight hours after transfection, cells were serum starved for 24 hours, and incubated further with or without HGF for the indicated time periods. Rac activity was determined using the Rac G-LISA Activation Assay as detailed in Materials and Methods. The results shown represent the means +/− SEM of at least 3 independent experiments (* = p < 0.05, two-tailed *t* test). **C**) depletion of RhoG does not affect expression of Rac1 nor *vice versa*. Cells were transfected with siRNAs (10 nM) directed against luciferase (control) or RhoG (two different oligos). Cells were lysed 72 hours after transfection, followed by western blot analysis. Histograms shows quantification (+/− SEM) of between 3 and 4 independent experiments.

To start dissecting the signaling mechanisms that are mediated by RhoG in glioblastoma, we first examined whether RhoG is activated by HGF (hepatocyte growth factor, also known as scatter factor). HGF is the most potent chemotactic factor known for glioblastoma cells [22]. In addition, expression levels of HGF and its receptor cMet correlate with astrocytoma grade [23,24]. Importantly, interference with cMet signaling, either using ribozymes targeting HGF or cMet, or a cMet-targeting small molecule, inhibits glioblastoma tumor growth *in vivo*[25,26]. Stimulation of SNB19 cells with HGF activates RhoG within 2 minutes and subsequently declines (Figure 2A).

RhoG has been shown to act upstream of the Rac1 small GTPase [27,28] and Rac1 is also known to be activated by HGF [29]. We therefore examined whether RhoG mediates HGF-induced Rac1 activation in glioblastoma cells. Rac1 activation was determined using an ELISA assay that quantifies the amount of Rac proteins in cell lysates through binding to an immobilized Rac effector [30]. Since Rac1 is the major Rac isoform expressed in glioma cells, this assay essentially reports on the activation state of Rac1 [10]. We found that HGF-induced Rac1 activation in SNB19 cells peaks at around 4 minutes, significantly slower than the kinetics of RhoG activation (Figure 2B). We also observed that depletion of RhoG significantly inhibits HGF-stimulated Rac1 activation. These results indicate that RhoG indeed functions upstream of Rac1 in HGF-stimulated cells, consistent with the observation that HGF-induced activation of RhoG precedes that of Rac1.

We also examined whether depletion of RhoG modulates the expression of Rac1, as this would complicate our analysis. We found that depletion of RhoG does not affect expression of Rac1 nor *vice versa*.

**Figure 3:**
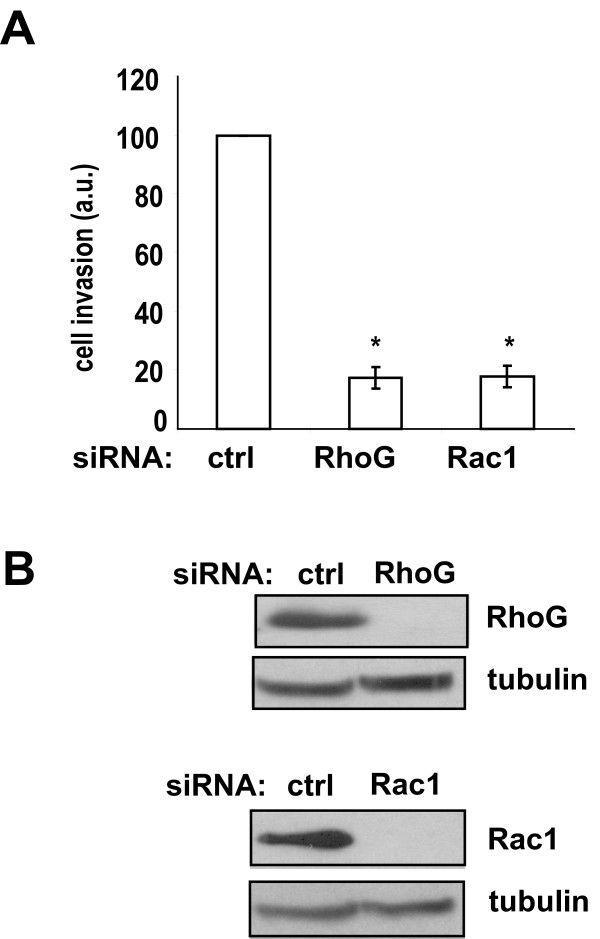
** RhoG mediates HGF-induced invasion. A**) SNB19 glioblastoma cells were transfected with siRNAs directed against luciferase (control, 20 nM), RhoG-2 (10 nM) or Rac1-1 (20 nM). Two days after transfection, cells were serum starved overnight and processed for the Matrigel invasion assay as described in Materials and Methods. All invaded cells on the bottom of the filter were counted using an inverted microscope. Data were normalized to those of control cells. The results shown represent the means +/− SEM of 4 independent experiments, performed in duplicate (* = p < 0.0005, two-tailed *t* test). **B**) Western blot demonstrating depletion of RhoG and Rac1.

Interestingly, depletion of RhoG only partially inhibits HGF-stimulated Rac1 activity (by approximately 40%), indicating the existence of RhoG-independent signaling mechanisms that contribute to Rac1 activation in this context. In contrast, depletion of RhoG largely abolishes HGF-induced invasion of SNB19 cells in a three-dimensional Matrigel invasion assay. This inhibitory effect is as large as that caused by depletion of Rac1 (Figure 3).

**Figure 4:**
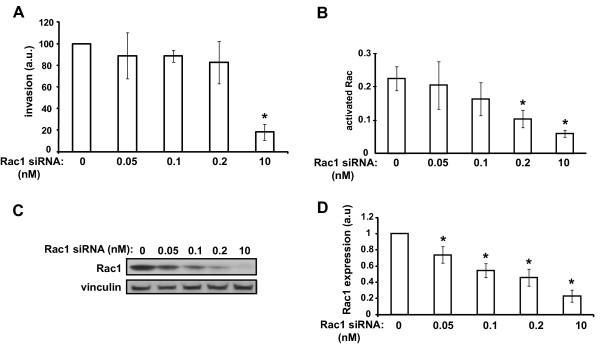
** Rac1-mediated invasion depends on a threshold of Rac1 expression. A**) Rac1 siRNA-concentration dependence of cell invasion. SNB19 cells were transfected with the indicated Rac1 siRNA concentrations and invasion assays were performed as in Figure 3. The results shown represent the means +/− SEM of 4 independent experiments, performed in duplicate. Data were normalized to those of control cells. **B**) Rac1 siRNA-concentration dependence of Rac1 activation state. Cells were transfected with the indicated Rac1 siRNA concentrations and Rac activity was determined as in Figure 2B. The results shown represent the means +/− SEM of 5 independent experiments. **C** and **D**) Rac1 siRNA-concentration dependence of Rac1 expression. **C**) Representative western blot demonstrating siRNA-dependent depletion of Rac1. **D**) Quantification of western blots for experiments shown in A) and B). The results shown represent the means +/− SEM of 8 independent experiments. Data were normalized to those of control cells.

To examine whether partial inhibition of HGF-stimulated Rac1 activation, similar to that caused by depletion of RhoG, could be sufficient to block HGF-induced invasion, we transfected SNB19 cells with a range of Rac1 siRNA concentrations. Interestingly, we observed that depletion of Rac1 by up to 60%, which inhibits Rac1 activation by at least 50%, does not cause significant inhibition of cell invasion (Figure 4). This implies that Rac1-mediated invasion depends on a threshold of Rac1 expression. Thus, taken together, the results shown in Figures 3 and 4 strongly suggest that RhoG regulates glioblastoma cell invasion via both Rac1-dependent and Rac1-independent mechanisms.

**Figure 5:**
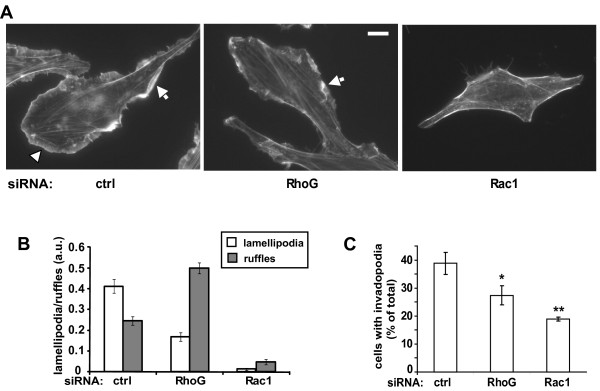
** RhoG is necessary for the formation of lamellipodia and invadopodia. A**) Fluorescence micrographs illustrating the effects of knockdown of RhoG and Rac1 on the formation of lamellipodia and ruffles. SNB19 glioblastoma cells were transfected with siRNAs directed against luciferase (control, 20 nM), RhoG-2 (10 nM) or Rac1 (20 nM). Two days after transfection, cells were plated on laminin-coated coverslips and serum starved overnight. Subsequently, cells were incubated with DMEM (untreated) or stimulated 4 min with DMEM containing 25 ng/ml HGF and processed as described in Materials and Methods. Scale bar represents 10 μm. Lamellipodia are indicated by an arrowhead. Ruffles are indicated by arrows. **B**) Quantification of lamellipodia and ruffle formation. Presented are mean values (+/− SEM) for approximately 35 cells per condition. The results shown are representative of two independent experiments. **C**) RhoG is necessary for invadopodia formation. SNB19 cells were transfected with siRNA (20 nM) directed against luciferase (control), RhoG-2 or Rac1-1. Forty eight hours after transfection, cells were split to a new dish for another 24 hours prior to plating on FITC-gelatin coated coverslips for 16 to 18 hours. Invadopodia formation was determined as described in Materials and Methods. For each condition, 16 (60x) fields were quantified, comprising a total of approximately 30 cells. The results shown represent the means +/− SEM of 5 independent experiments (*p < 0.05 and **p < 0.0005, two-tailed *t* test).

To further explore the mechanisms that mediate RhoG-regulated glioblastoma cell invasion, we examined the role of RhoG in the formation of two structures that have been implicated in Rac1-regulated cell migration and invasion: invadopodia and lamellipodia. We also compared depletion of RhoG with that of Rac1 under the same conditions. Whereas depletion of Rac1 completely abrogates HGF-induced lamellipodia formation in SNB19 cells, as observed previously for serum-stimulated lamellipodia formation in these cells [10], depletion of RhoG has a much smaller inhibitory effect on this function (Figure 5A, B). Surprisingly, we noted that the extent of membrane ruffling, as defined by lamellipodia that lift off from the substrate and fold back toward the center of the cell, is actually stimulated by depletion of RhoG, whereas depletion of Rac1, as expected, strongly inhibits ruffle formation.

Depletion of RhoG significantly inhibits invadopodia formation, but less efficiently than depletion of Rac1 (Figure 5C). Taken together, the observations that depletion of RhoG strongly inhibits glioblastoma cell invasion, but only partially inhibits the formation of invadopodia and lamellipodia, further supports the conclusion that RhoG regulates glioblastoma cell invasion using both Rac1-dependent and -independent mechanisms.

### RhoG mediates EGF-stimulated Rac1 activation

**Figure 6:**
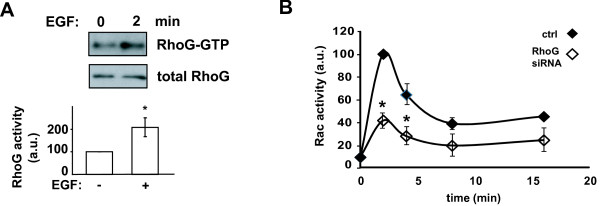
** RhoG mediates EGF-induced Rac1 activation. A**) RhoG is activated by EGF (50 ng/ml) in SNB19 cells. Western blot shows activated RhoG and total RhoG for HGF-stimulated and controls cells. Histogram shows quantification (+/− SEM) of 5 independent experiments (p < 0.05, two-tailed *t* test). **B**) RhoG mediates EGF-induced Rac activation. Cells were transfected with siRNAs (10 nM) directed against luciferase (control) or RhoG-2. Forty eight hours after transfection, cells were serum starved for 24 hours, and incubated further with or without EGF for the indicated time periods. Rac activity was determined as in Figure 2B. The results shown represent the means +/− SEM of 3 independent experiments (* = p < 0.05, two-tailed *t* test).

A recent study has demonstrated that RhoG is activated downstream of the EGF receptor (EGFR) [19]. Since EGFR is amplified and/or mutated in 45% of all glioblastoma tumors [31,32], we also examined whether RhoG mediates EGF-stimulated signaling in glioblastoma cells. We observed that EGF (50 ng/ml) significantly activates RhoG in SNB19 cells (Figure 6A). In addition, depletion of RhoG significantly inhibits EGF-stimulated Rac1 activation in these cells (Figure 6B).

### RhoG promotes glioblastoma cell survival

**Figure 7:**
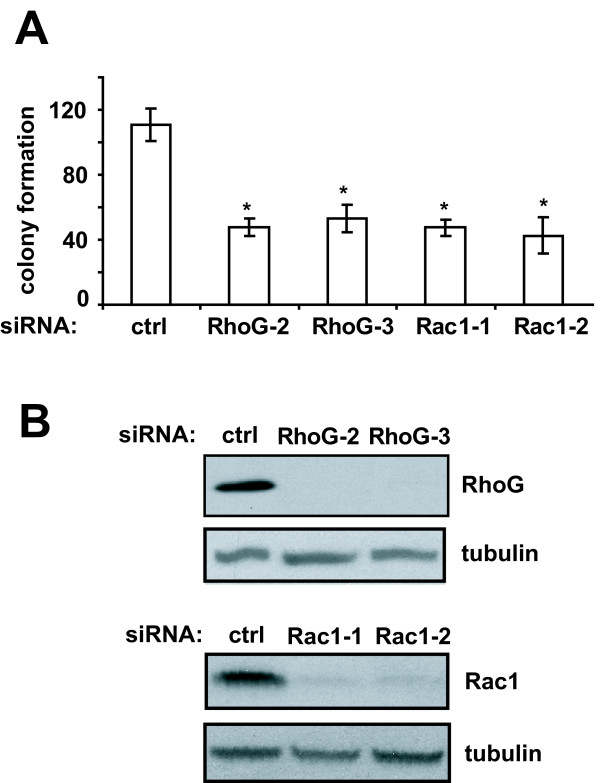
** RhoG and Rac1 contribute to glioblastoma cell clonogenicity. A**) Colony formation assay. SNB19 cells were transfected with siRNAs (10 nM) directed against luciferase (control), RhoG or Rac1. Colony formation assays were performed as described in Materials and Methods. The results shown represent the means +/− SEM of 4 independent experiments (each performed in quadruplet dishes) for the RhoG and 3 independent experiments for the Rac1 knock-downs (p < 0.001, two-tailed *t* test). **B**) Western blot demonstrating depletion of RhoG and Rac1.

Previous studies have implicated RhoG in the regulation of cell survival through direct binding to the regulatory subunit of PI3K and subsequent activation of Akt [17,33]. We therefore also examined the role of RhoG in glioblastoma cell survival using a clonogenicity assay. We observed that depletion of RhoG strongly inhibits glioblastoma cell colony formation, similar to the extent of depleting Rac1 (Figure 7), indicating a significant role for RhoG in glioblastoma cell survival.

We also examined whether depletion of RhoG sensitizes glioblastoma cells to ionizing radiation, which is part of the current standard protocol for glioblastoma therapy. We found that the inhibitory effect of depleting RhoG on cell survival is additive to that of ionizing radiation, rather than showing synergism (data not shown), indicating that RhoG does not specifically contribute to radio-resistance.

### RhoG is overexpressed in human glioblastoma tissue

**Figure 8:**
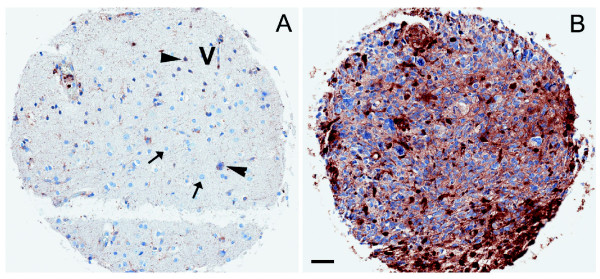
** RhoG is strongly expressed in glioblastoma tumors. A**) Representative RhoG immunohistochemistry micrograph of reactive tissue adjacent to tumor. “V” indicates vessel, arrows indicate normal glial cells and arrowheads point to reactive astrocytes in the tumor rim. **B**) Representative RhoG immunohistochemistry micrograph of tumor core. Scale bar represents 100 μm.

Available microarray expression data, such as NCBI dataset GSE4290, did not reveal any significant change of RhoG mRNA expression levels across non-neoplastic brain and different grades of astrocytoma (data not shown). However, immunohistochemical analysis revealed prominent cytoplasmic RhoG staining (with scores 2–3 out of 3) in glioblastoma cells in all 41 tumor samples tested (Figure 8B). Staining is also visible in some reactive astrocytes (Figure 8A, arrowhead), but not in non-reactive non-neoplastic glial cells (Figure 8A, arrow). Significant expression is also observed in endothelial cells of the tumor microvasculature (Figure 8A). However, using an astrocytoma progression tissue microarray, we did not observe significant differences between the staining intensity of tumor cells across different astrocytoma grades, including pilocytic, grade II, anaplastic and grade IV astrocytomas (data not shown).

## Discussion

In this paper, we show that the small GTPase RhoG plays a critical role in glioblastoma cell invasion, both in *in vitro* and *ex vivo* settings. In addition, RhoG is important for glioblastoma cell survival, but less so for proliferation. Thus, RhoG contributes significantly to the malignant behavior of glioblastoma cells.

We also found that RhoG is activated by cMet and EGFR, two receptors that are deregulated in glioblastoma tumors [31,32,34], further underlining the relevance of RhoG-mediated signaling in the context of glioblastoma. Compared to other Rho family members, relatively few receptors that stimulate RhoG activity have been identified to date. These include ICAM-1 and syndecan 4 [35,36]. RhoG signaling also is thought to mediate signaling from the fibronectin receptor [28]. Recently, EGFR also has been shown to activate RhoG [19]. Here, we confirm this finding in a clinically relevant setting and add cMet to the list of receptors that stimulate RhoG activity.

Early observations have indicated the existence of both Rac1-dependent and Rac1-independent functions of RhoG [27,37]. A subsequent key finding was that RhoG-stimulated activation of Rac1 is mediated by direct interaction of RhoG with ELMO [28]. ELMO forms a stable complex with the Rac GEF Dock180 that in turn activates Rac1 and regulates neurite outgrowth [28,38]. Moreover, genetic studies using C. elegans have provided evidence that the RhoG/ELMO/Dock180/Rac1 module is evolutionary conserved and mediates phagocytosis of apoptotic cells [39]. RhoG also acts upstream of Rac1 in the regulation of cell migration [18].

Here, we show that depletion of RhoG significantly inhibits both HGF- and EGF-stimulated Rac1 activation in glioblastoma cells, demonstrating that RhoG acts upstream of Rac1 in these cells. Our finding that RhoG contributes to Rac1 activation downstream of EGFR in glioblastoma cells however, contrasts to published observations that EGF-stimulated Rac1 activation is independent of RhoG in HeLa cells [19]. These differences may reflect cell type-specific differences in signaling.

The inhibitory effect of RhoG depletion on Rac1 activation is only partial (approximately 40% and 60% inhibition for respectively HGF- and EGF-induced Rac1 activity), indicating that cMet and EGFR also can activate Rac1 in a RhoG-independent fashion. Interestingly however, in spite of the fact that RhoG only partially contributes to HGF-induced Rac1 activation, the inhibitory effect of depleting RhoG on glioblastoma cell invasion is as large as that of depleting Rac1, indicating that RhoG regulates glioblastoma cell invasion in both Rac1-dependent and Rac1-independent ways. A Rac1-independent role of RhoG in cell migration also has been demonstrated in fibroblasts derived from Rac1 knockout mice [40]. RhoG directly binds to the p85 regulatory subunit of PI3K, leading to the activation of Akt [17,33], presenting a possible mechanism for Rac1-independent stimulation of invasion by RhoG. However, we did not observe a significant effect of depleting RhoG on HGF-stimulated Akt activation (data not shown), suggesting the existence of additional RhoG-controlled signaling mechanisms.

Consistent with our observations that RhoG contributes to Rac1 activation by HGF, we also observed that depletion of RhoG partially inhibits HGF-induced formation of lamellipodia. Surprisingly however, depletion of RhoG enhances the formation of membrane ruffle formation, whereas depletion of Rac1 strongly inhibits ruffling. RhoG is thought to mediate integrin signaling and depletion of RhoG has been shown to slow down cell spreading [18,28], which we have confirmed in glioblastoma cells (data not shown), suggesting that a decrease in cell adhesion in the RhoG-depleted glioblastoma cells may be responsible for this increase in membrane ruffling.

Previous data from our laboratory have demonstrated that Rac1 is critical for glioblastoma cell proliferation in the absence, but not in the presence of serum [10]. Somewhat surprisingly, our current results do not reveal an important role for RhoG in gliobastoma cell proliferation, neither in the absence nor in the presence of serum. This suggests that partial inhibition of Rac1 activation is not sufficient to affect Rac1’s ability to stimulate cell proliferation. Interestingly, RhoG has been implicated in the regulation of the proliferative behavior of neural progenitor cells [21]. The stimulatory effect of RhoG on neural progenitor cell proliferation depends on PI3K activation, but not on the interaction of RhoG with ELMO, suggesting that this effect is independent of RhoG-mediated activation of Rac1.

Several observations in the literature have shown a strong correlation between tumor cell invasiveness and survival potential, both in glioblastoma [41,42] and breast cancer [43], suggesting the existence of signaling nodes that coordinate tumor cell invasion with survival. Thus, our observations that both RhoG and Rac1 significantly inhibit glioblastoma cell colony formation, identify these GTPases as such signaling nodes. We hypothesize that these invasion/survival signaling nodes present attractive targets for therapeutic intervention.

## Conclusions

Our functional analysis of RhoG in the context of glioblastoma has revealed a critical role for RhoG in tumor cell invasion and survival. Furthermore, our findings that RhoG is overexpressed in human glioblastoma tumors and that this GTPase is activated by signaling pathways that are hyperactive in glioblastoma tumors, provide a strong indication for the use of RhoG and elements of RhoG-regulated signaling pathways as novel drug targets for the treatment of glioblastoma. It will be interesting to see whether these findings extend to other tumor types. Importantly, RhoG, in contrast to Rac1, appears to play restricted roles in normal cells, which is supported by the fact that RhoG knockout mice are developmentally normal and display only minor immunological phenotypes [44]. This suggests that inhibitors of RhoG signaling should display a relatively large therapeutic window. Notably, GTPases are thought to be challenging drug targets [45]. However, over the past several years, a number of different avenues have opened up, mostly focusing on the Rac1 GTPase as a target, including structure-based virtual screening, the development of peptidic structures and the implementation of siRNA delivery [46-52]. This wide range of approaches is also applicable to RhoG, and it is hoped that future efforts will yield RhoG-specific inhibitors.

## Methods

### siRNAs

Two siRNA duplexes targeting RhoG were used, a 21 nucleotide siRNA with target sequence 5'- GCAACAGGATGGTGTCAAG-3' [35] was purchased from Ambion and a 27 nucleotide siRNA with target sequence 5'- CACUUCCUUGACACCAUCCUGUUGCAG' was purchased from Integrated DNA Technologies. A control siRNA directed against GL2 luciferase with target sequence 5'- AACGTACGCGGAATACTTCGATT was purchased from Ambion. The Rac1 siRNAs have been described previously [10]. Depletion of the respective proteins was confirmed by western blotting using monoclonal antibodies against Rac (clone 23A8, Upstate Biotechnology) and RhoG (clone 1 F3 B3 E5, Millipore). A monoclonal antibody against α-tubulin (Sigma) was used as loading control.

### Cell culture and siRNA transfections

Cells were grown at 37°C in the presence of 5% CO_2_ in DMEM supplemented with 10% FBS and penicillin/streptomycin. Transient transfection of siRNA was carried out with Dharmafect (Dharmacon) solution #1 at 1 μl/ml, using the protocol provided by the manufacturer. Cells were assayed, typically 3–4 days after transfection, when maximal knockdown was observed.

### RhoG activity assay

RhoG activity was measured essentially as described previously [53]. GST-ELMO-NT (GST fusion protein containing the N-terminal RhoG-binding domain of ELMO2, amino acids 1– 362) was expressed in Escherichia coli (BL21) and purified using a B-PER GST fusion protein purification kit (Thermo scientific). Purified GST-ELMO NT was stored in 100 mM Tris–HCl, pH 7.5, 2 mM MgCl_2_ and 0.1 mM DTT, at −80°C. To measure RhoG activity, SNB19 cells grown in 10 cm dishes were serum starved for 24 hours and stimulated with either 25 ng/ml HGF or 50 μg/ml EGF for various times. Subsequently, cells were washed with 10 ml of cold PBS and lysed in ice-cold cell lysis buffer (50 mM Tris–HCl, pH 7.5, 100 mM NaCl, 10 mM MgCl_2_, 1% Triton X-100, 10% glycerol, 1 mM DTT and protease inhibitor cocktail (Roche). Cell lysates were centrifuged for 2 minutes at 13,000 *g* at 4°C. For each condition, 500 mg of supernatant was incubated with 20 μg of GST-ELMO-NT and 10 μl of GST beads for 1 hour at 4°C. The beads were washed with lysis buffer and bound proteins were analyzed by western blotting using a monoclonal anti-RhoG antibody (Millipore). Semi-quantification of RhoG activity was performed by gel-scanning, determining the amount of RhoG bound to GST-ELMO-NT normalized to the amount of total RhoG present in cell lysates.

### Rac activation assay

Rac activation was determined using the Rac G-LISA Activation Assay (Cytoskeleton) according to the manufacturer’s recommendations. In brief, SNB19 cells were transfected with control or RhoG siRNA. 48 hours after transfection, cells at 60-70% confluency were serum starved for 24 hours and treated with either HGF or EGF. Subsequently, cells were washed with ice-cold PBS, followed by lysis at 4°C. Cell lysates were clarified by centrifugation at 13,000 *g* at 4°C for 2 minutes and snap frozen in liquid nitrogen. For each condition, 50 μl of cell lysate (containing 25 μg of protein) was added to a single well of the Rac GTPase binding plate. Further steps were carried out exactly as described in the protocol provided by the manufacturer.

### Brain slice invasion assay

The brain slice invasion assay was performed as described previously [20], with minor modifications. In brief, 24 hours after transfection, GFP-labeled cells were deposited bilaterally onto the putamen of 400 μm thick slices of freshly isolated 4–6 week-old mouse brains. Serial Z-sections were collected by confocal laser scanning microscopy and the extent of invasion was determined as the maximum depth of invasion of the glioblastoma cells.

### Matrigel invasion assay

First, the bottom side of the filter (8 μm pore size) was coated with a thin layer of fibronectin by overlaying with a drop of fibronectin (1 μg/ml) for 1 hour at room temperature, followed by rinsing with PBS. Subsequently, 9 x 10^4^ glioma cells were suspended in 50 μl of Matrigel kept at 4°C (10 mg/ml) (Trevigen) and added to the top well (insert) of a 24-well transwell plate (BD). The matrix was allowed to solidify at 37°C for 30 minutes. Next, 200 μl of serum-free DMEM medium was added to the top well and 700 μl of serum-free DMEM containing HGF (25 ng/ml) to the bottom well. After 24 – 48 hours of incubation, the insert was fixed in 4% formaldehyde/PBS and stained with crystal violet. Subsequently, cells on the upper surface of the filter were wiped off with a Q-tip and cells attached to the bottom side of the filter counted under the microscope (Olympus IX70, 20x objective).

### Quantification of lamellipodia formation

Serum-starved cells plated on laminin–covered coverslips (Becton Dickinson) were incubated with DMEM (control) or stimulated for 4 minutes with DMEM containing 25 ng/ml HGF. Subsequently, cells were fixed in 4% formaldehyde/PBS, permeabilized with 0.1% Triton-X100 dissolved in PBS and incubated with Rhodamine-conjugated phalloidin (Molecular Probes) to stain for F-actin. Processed coverslips were mounted in 75% Vectashield mounting medium (Vector Laboratory). Images were collected using a Zeiss Axiovert 200 M microscope, equipped with a 63x objective, a cooled CCD camera (AxioCam monochromatic) and Axiovision 4.7 image analysis software. For each experimental condition, images were taken in a random fashion. Lamellipodia and ruffles were traced using Image J software. For each cell, the fraction of the cell perimeter that displays either lamellipodia or ruffles was calculated.

### Sulphorhodamine B assay

Cell proliferation was measured using the sulphorhodamine B colorimetric assay [54]. Briefly, one day after siRNA transfection, cells were seeded in septuplet in 96-well microtiter plates in serum-containing medium at 2.5 x 10^3^ cells/well for determining growth in the presence of serum and at 5 x 10^3^ cells/well for determining growth in the absence of serum. Once the cells attached and spread (5 hours after plating), for a subset of the wells, the medium was replaced with serum-free medium. At various times, cells were fixed in 10% trichloroacetic acid for 1 hour at 4°C, rinsed and subsequently stained for 30 minutes at room temperature with 0.2% SRB dissolved in 1% acetic acid, followed by air drying. The bound dye was solubilized in 200 μl of 10 mM unbuffered Tris base for 30 minutes and the OD was read at 490 nm in an ELISA plate reader.

### Invadopodia formation assay

FITC-gelatin coated coverslips were prepared as described previously [55], with minor modifications. Briefly, in a 20 mg/ml gelatin solution (300 bloom gelatin in 50 mM Na_2_B_4_O_7_/40 mM NaCl, pH 9.3), FITC (Sigma-Aldrich, St. Louise, MO) was added to a 0.2 mg/ml final concentration for two hours, followed by dialysis against PBS for 2 to 3 days at 37°C. Thin layers of FITC–conjugated gelatin were placed on coverslips, cross-linked with 0.8% glutaraldehyde for 10 minutes on ice, followed by 30 minutes at room temperature. Subsequently, coverslips were incubated with 5 mg/ml of sodium borohydride for 3 minutes and sterilized with 70% ethanol for 10 minutes. Sterilized FITC-gelatin coated coverslips were left dry for at least 15 minutes in the tissue culture hood. One hour before plating the cells, coverslips were re-hydrated with DMEM containing 10% fetal bovine serum at 37°C. Cells were cultured on the FITC-gelatin coverslips in DMEM, supplemented with 0.5% FBS, for 16 to 18 hours. Subsequently, the cells were fixed and processed for immunofluorescence as described in the section on quantification of lamellipodia formation. Invadopodia formation was determined by counting the percentage of cells that had an associated area of matrix degradation.

### Colony formation assay

Forty eight hours after transfection, SNB19 cells were seeded in 6 cm plates at a density of 300 cells per dish. The cells were incubated for 12 days, with medium changes every 3 or 4 days. Plates were fixed in formaldehyde solution (4%) and colonies were stained with sulphorhodamine B (see above). The plates were imaged using a scanner and colonies were counted using Adobe Photoshop CS3 Extended Edition or ImageJ, with a threshold that corresponds to 50 cells/colony.

### Immunohistochemistry

The tissue microarray and immunohistochemistry procedures that we have used to examine RhoG expression in the rim and core of glioblastoma tumors have been described previously [56].

## Abbreviations

CCD, Charge-coupled device; DAPI, 4',6-diamidino-2-phenylindole; DMEM, Dulbecco’s Modified Eagle Medium; DTT, Dithiothreitol; EGF, Epidermal growth factor; EGFR, EGF receptor; ELMO, Engulfment and Cell Motility; FITC, Fluorescein isothiocyanate; GAP, GTPase activating protein; GEF, Guanine nucleotide exchange factor; GFP, Green fluorescent protein; GST, Glutathione S-transferase; HGF, Hepatocyte growth factor; OD, Optical density; PBS, Phosphate-buffered saline; PI3K, Phosphatidylinositol 3’-kinase; siRNA, Small interfering RNA.

## Competing interests

The authors declare that they have no competing interests.

## Authors’ contributions

AK performed the cell proliferation, *in vitro* invasion and lamellipodia formation studies and participated in the Rac activation assays. SD performed the RhoG and Rac activation studies and part of the colony formation assays. SF performed the brain slice invasion assay. YC performed the invadopodia formation assay. TW performed part of the colony formation assays. MEB provided the TMA slides and ER and JE interpreted and scored the TMA assays. NT supervised and coordinated performance of the brain slice assay and IHC staining. AC participated in the design of the study, implemented the RhoG activation assay and produced preliminary data for most of the functional assays. MS designed and coordinated the overall study and drafted the manuscript. All authors read and approved the final manuscript.
